# Efficacy and safety of combined targeted therapy and immunotherapy versus targeted monotherapy in unresectable hepatocellular carcinoma: a systematic review and meta-analysis

**DOI:** 10.1186/s12885-022-10174-6

**Published:** 2022-10-21

**Authors:** Teng-Kai Yang, Ya-Fang Yu, Chiao-Ling Tsai, Hsing-Ju Li, Po-Sheng Yang, Kai-Wen Huang, Jason Chia-Hsien Cheng

**Affiliations:** 1 Department of Surgery, Yonghe Cardinal Tien Hospital, New Taipei City, Taiwan; 2 School of Medicine, College of Medicine, Fu-Jen Catholic University, New Taipei, Taiwan; 3 Graduate institute of Clinical Medicine, National Taiwan University College of Medicine, Taipei, Taiwan; 4 Division of Radiation Oncology, Department of Oncology, National Taiwan University Hospital, No. 7, Chung-Shan South Road, 100225 Taipei, Taiwan; 5 Department of General Surgery, Mackay Memorial Hospital and Mackay Medical College, Taipei, Taiwan; 6 Department of Surgery, National Taiwan University Hospital, Taipei, Taiwan; 7 Graduate Institute of Oncology, National Taiwan University College of Medicine, Taipei, Taiwan

**Keywords:** Targeted therapy, Immunotherapy, Unresectable hepatocellular carcinoma, Systematic review, Meta-analysis

## Abstract

**Background:**

Cancer therapy has evolved from non-specific cytotoxic agents to a selective, mechanism-based approach that includes targeted agents and immunotherapy. Although the response to targeted therapies for unresectable hepatocellular carcinoma (HCC) is acceptable with the improved survival, the high tumor recurrence rate and drug-related side effects continue to be problematic. Given that immune checkpoint inhibitor alone are not robust enough to improve survival in unresectable HCC, growing evidence supports the combination of targeted therapy and immunotherapy with synergistic effect.

**Methods:**

Online databases including PubMed, EMBASE, Cochrane Library, and Web of Science were searched for the studies that compared targeted monotherapy with the combination therapy of targeted drug and checkpoint inhibitors in unresectable HCC patients. Eligibility criteria were the presence of at least one measurable lesion as defined by the Response Evaluation Criteria in Solid Tumors (version 1.1) for unresectable HCC patients, an Eastern Cooperative Oncology Group performance status of 0–2, and a Child–Pugh score ≤ 7. Outcome measurements include overall survival (OS), progression-free survival (PFS), and treatment-related adverse event (TRAE).

**Results:**

Three phase II/III randomized controlled trials were included in this study. The pooled results showed that combination therapy significantly improved survival than targeted monotherapy, in terms of OS (hazard ratio (HR) = 0.67; 95% confidence interval [CI]: 0.50–0.91) and PFS (HR = 0.58; 95% CI: 0.51–0.67), respectively. In the incidence of grade 3–5 TRAEs, the combination therapy was significantly higher than targeted monotherapy (odds ratio = 1.98; 95% CI: 1.13–3.48).

**Conclusion:**

For unresectable HCC, combined targeted drug and immunotherapy significantly improved survival compared with targeted monotherapy. However, the incidences of AEs of combinational therapy were higher than targeted monotherapy.

## Introduction

Cancer is one of the important global health issues, which claims the live of approximately one in six individuals. In 2020, an estimated 19.3 million new cases of cancer and nearly 10 million cancer-related deaths were reported worldwide [1, 2].

For many decades, there have been various options of cancer treatment for patients, including surgery, radiation therapy, and chemotherapy, either alone or in combination. In the last three decades, medical research has advanced substantially in the molecular understanding of cancer biology. From relatively non-specific cytotoxic agents to a specifically selective, mechanism-based approach, including targeted agents and cancer immunotherapy, cancer therapy has evolved [1, 3]. This approach has been used for a wide range of solid tumors including hepatocellular carcinoma (HCC) [4–6].

The mechanistic action of targeted therapy is by interfering with specific molecules, which blocks the growth and spread of cancer. Although the initial response to targeted therapies was acceptable with the improved survival in a proportion of patients, obstacles exist with the high rate of tumor recurrence and drug-related side effects. Targeted therapies remain common in treating patients with unresectable and advanced HCC [3, 7, 8], so the need for more effective and safer alternative therapies is urgently warranted.

Immunotherapy aims to stimulate a host immune response that destructs tumor and enhances antitumor responses to inhibit tumor growth or kill cancer cells [1, 9]. In patients with unresectable HCC, monotherapy with immune checkpoint inhibitors was not robust enough to improve overall survival (OS) and/or progression-free survival (PFS) [10, 11]. However, there is growing evidence that the combination of targeted therapy and immunotherapy has the potential to provide synergistic and sustained effects for cancer management [12, 13] and for unresectable HCC [14, 15]. One network meta-analysis compared the efficacy and safety of all first-line systemic therapy in patients with unresectable HCC, and one of the results showed that checkpoint inhibitor plus targeted therapy provided better outcomes of OS and PFS than sorafenib [16]. Therefore, this systematic review and meta-analysis are aimed to evaluate the efficacy and safety of the combination therapy versus targeted monotherapy in patients with unresectable/advanced HCC.

## Methods

### Data source and literature search strategy

The search databases, including PubMed, EMBASE, Cochrane Library, and Web of Science, were searched for eligible studies from inception to July 2022. The search terms used to define the therapy included (“molecular targeted therapy” OR “targeted therapy”) AND (“immunotherapy” OR “immune checkpoint inhibitors” OR “programmed death 1 receptor” OR “programmed death 1 ligand 1” OR “PD 1 Inhibitors” OR “PD L1 Inhibitors”). The terms used to define the disease included “liver cell carcinoma” OR “advanced hepatocellular carcinoma” OR “hepatocellular carcinoma cell line.” In addition, we also checked the reference lists of all relevant articles to identify additional studies.

### Study selection

The inclusion criteria were as follows: [1] prospective study and randomized controlled trials (RCTs); [2] study involving patients with advanced/unresectable HCC; [3] intervention and comparison with targeted therapy in combination with PD-1/PD-L1 inhibitors compared with targeted monotherapy; [4] the presence of at least one measurable lesion as defined by the Response Evaluation Criteria in Solid Tumors (version 1.1); 5) Eastern Cooperative Oncology Group performance status of 0–2 in HCC patients; [6] Child–Pugh score ≤ 7; [7] at least one of the following clinical outcomes reported—OS, PFS, and the rate of any grade adverse events (AEs); and [8] studies published in English. The exclusion criteria were as follows: review articles, case reports, and conference abstracts.

### Data extraction and quality assessment

For each eligible study, the following information was extracted: article title, first author, publication year, trial phase, study design, applied agents, combination therapy, sample size, rate of OS, rate of PFS, median time to progression, AEs, and national clinical trial identification number. The risk of bias for individual studies was assessed at the study level based on the Cochrane Collaboration’s tool for randomized trials, which include the following domains: random sequence generation, allocation concealment, blinding, incomplete outcome data, and selective outcome reporting [16]. The evaluation of the risk of bias was conducted by the Review Manager (RevMan, V.5.4.1, Nordic Cochrane Centre, Cochrane, Copenhagen, Denmark).

### Statistical analysis

We calculated the pooled hazard ratio (HR) and 95% confidence interval (CI) for PFS and OS, as well as the pooled odds ratio (OR) and 95% CI for grade 3–5 TRAEs. The meta-analysis was conducted using the random-effects model under the assumption of significant heterogeneity. Heterogeneity among studies was quantified by I2 test, and I2 > 50% was considered substantial heterogeneity. p < 0.05 was considered statistically significant. The statistical analysis was conducted using Review Manager (RevMan5.4.1).

## Results

### Study selection and characteristics of the included studies

**Fig. 1:**
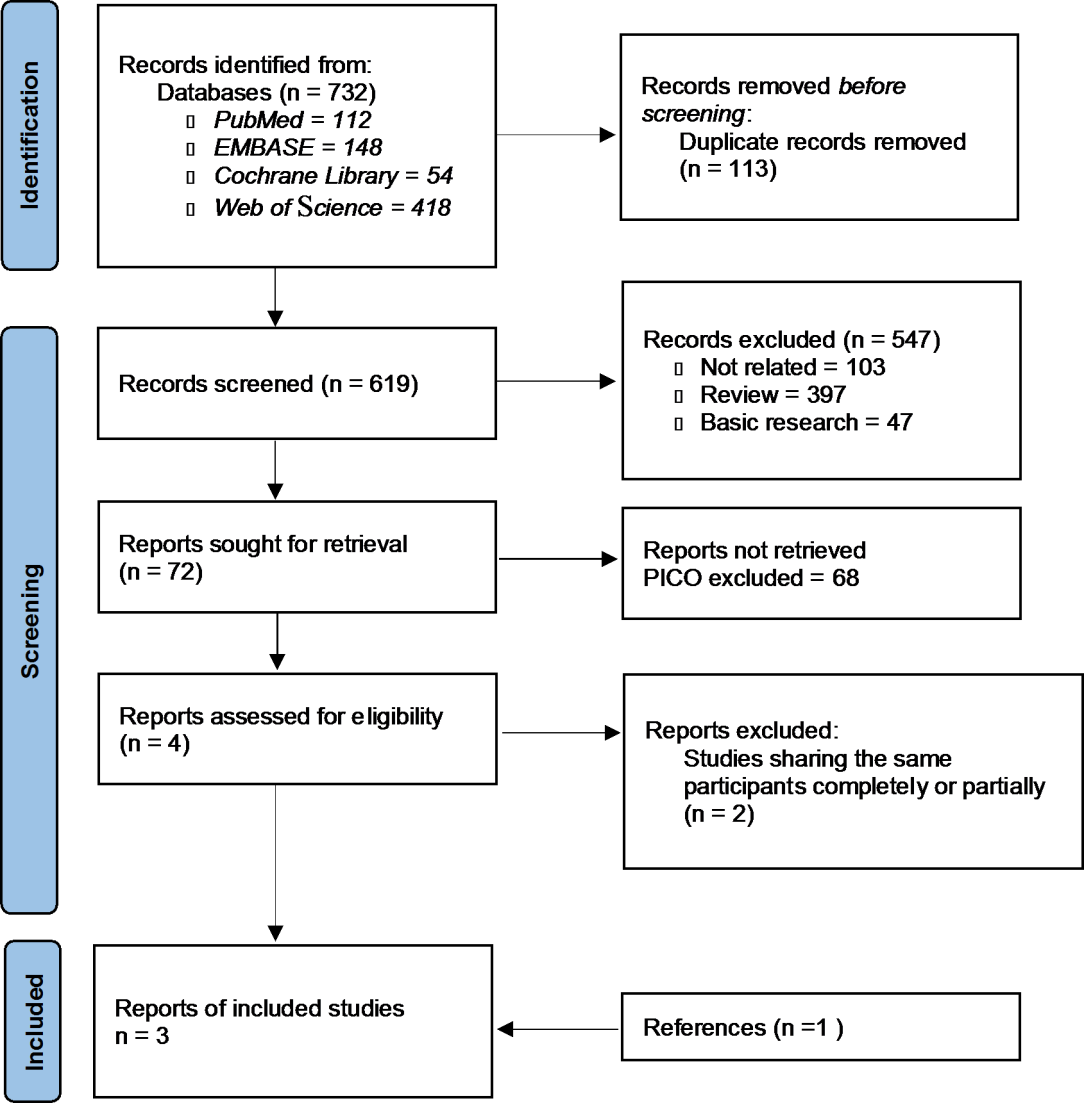
PRISMA flow diagram showing screening and selection process. Reference: Page, M. J., McKenzie, J. E., Bossuyt, P. M., Boutron, I., Hoffmann, T. C., Mulrow, C. D., et al. (2021). The PRISMA 2020 statement: an updated guideline for reporting systematic reviews. *International Journal of Surgery*, *88*, 105,906

The initial search identified 732 articles in online databases. After the screening process, duplicate and irrelevant studies were excluded. Finally, three articles were included in this meta-analysis (Fig. 1) [14, 15, 17]. Study designs for all studies were phase II/III RCTs. The studies were all published from 2020 to 2022. A total of 1,721 patients were included in the meta-analysis. The mean age was approximately 61 year, with a range from 53 to 66 year (Table 1).

**Table 1:** Baseline characteristics of the included studies

Types of Cancer	Study Name	Study phase/ design	Arm	Pts	Median Age	Male (%)	Median OS (m)	HR, 95%CI	Median PFS (m)	HR, 95%CI	TRAEs of grade 3–5	NCT number.
HCC	Finn et al.,2020 (IMbrave150)	III/RCT	Atezolizumab + Bevacizumab	336	64	277(82)	NE	0.58, 0.42–0.79	6.8	0.59, 0.47–0.76	201	NCT03434379
			Sorafenib	165	66	137(83)	13.2		4.3		95	
	Ren et al.,2021 (ORIENT-32)	III/RCT	Sintilimab + Bevacizumab biosimilar	380	53	334(88)	NR	0.57, 0.43–0.75	4.6	0.56, 0.46–0.70	231	NCT03794440
			Sorafenib	191	54	171(90)	10.4		2.8		68	
	Kelley et al.,2022 (COSMIC-312)	III/RCT	Atezolizumab + Cabozantinib	432	64	360(83)	15.4	0.90 0.69–1.18	6.8	0.63 0.44–0.91	236	NCT03755791
			Sorafenib	217	64	186(86)	15.5		4.2		68	

HCC = hepatocellular carcinoma; RCT = randomized control trial; OS = overall survival; CI = confidence interval; PFS = progression-free survival; HR = hazard ratio; AEs = adverse events; NR = not reached; NE = not estimable

### Risk of bias

**Fig. 2:**
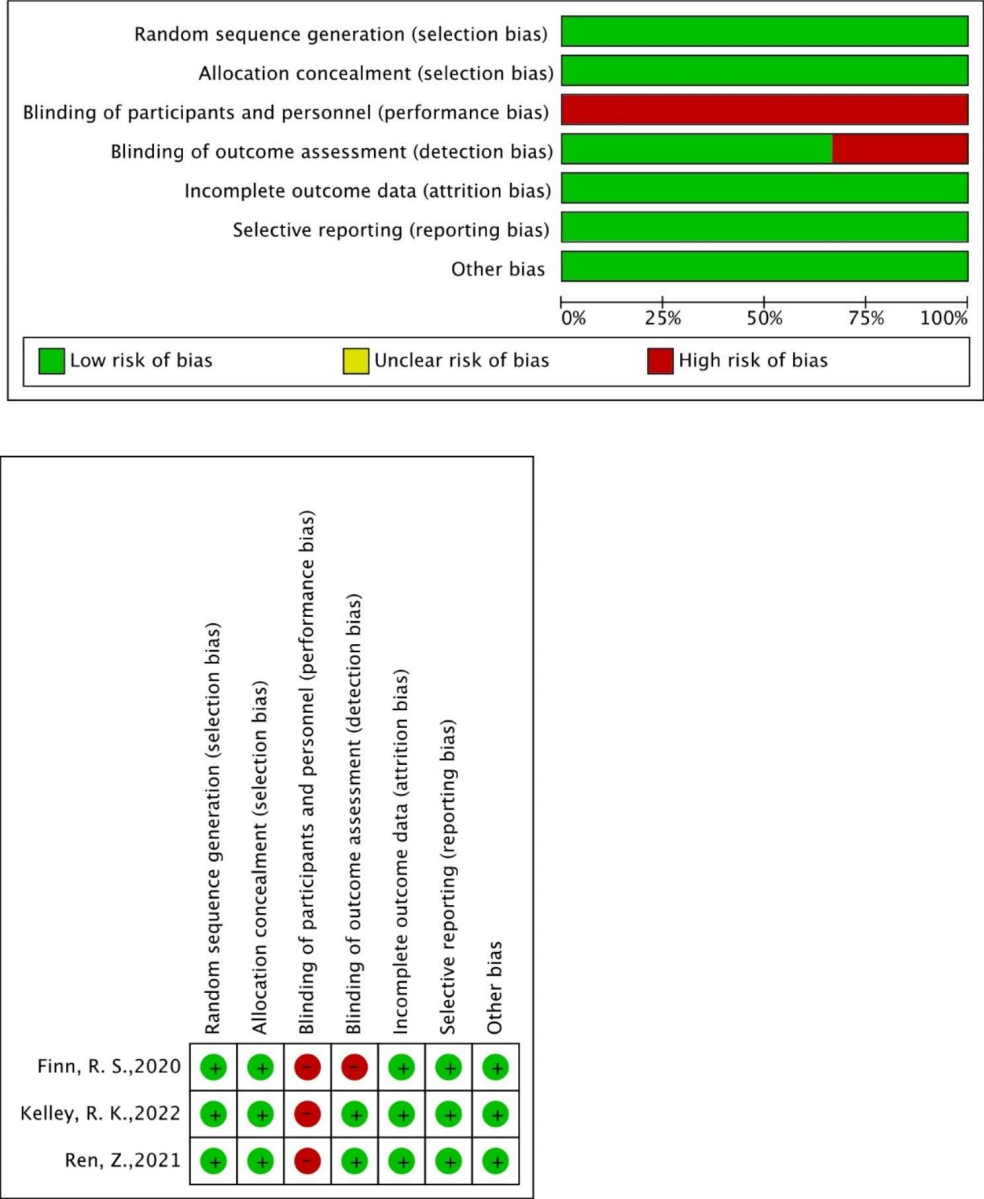
Risk of bias graph: with review of authors’ judgements about each risk of bias item presented as percentages across all included studies

Four domains of the included studies were found to have a low risk of bias (random sequence generation, allocation concealment, incomplete outcome data, and selective outcome reporting). All three studies rated the high risk of bias for blinding participants and personnel blinding bias. One study was rated as high risk for the blinding of outcome assessment (Fig. 2).

### Major outcomes: overall survival and progression-free survival

**Fig. 3:**
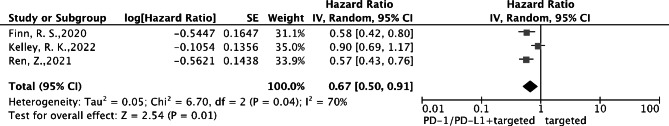
Forest plot of hazard ratio of overall survival using a random-effects model of hepatocellular carcinoma

OS and PFS data were available for all three trials. The pooled results showed that patients receiving combination therapy with targeted drug and immunotherapy had significantly better pooled OS than targeted monotherapy (HR = 0.67; 95% CI: 0.50–0.91) (Fig. 3).

**Fig. 4:**
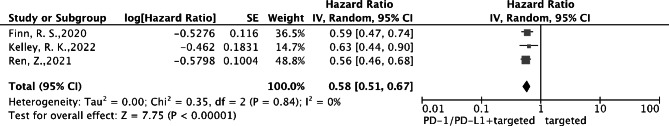
Forest plot of hazard ratio of progression-free survival using a random-effects model of hepatocellular carcinoma

For PFS, patients receiving combination therapy had significantly better pooled PFS than targeted monotherapy (HR = 0.58; 95% CI: 0.51–0.67) (Fig. 4).

### Treatment-related adverse events

**Fig. 5:**
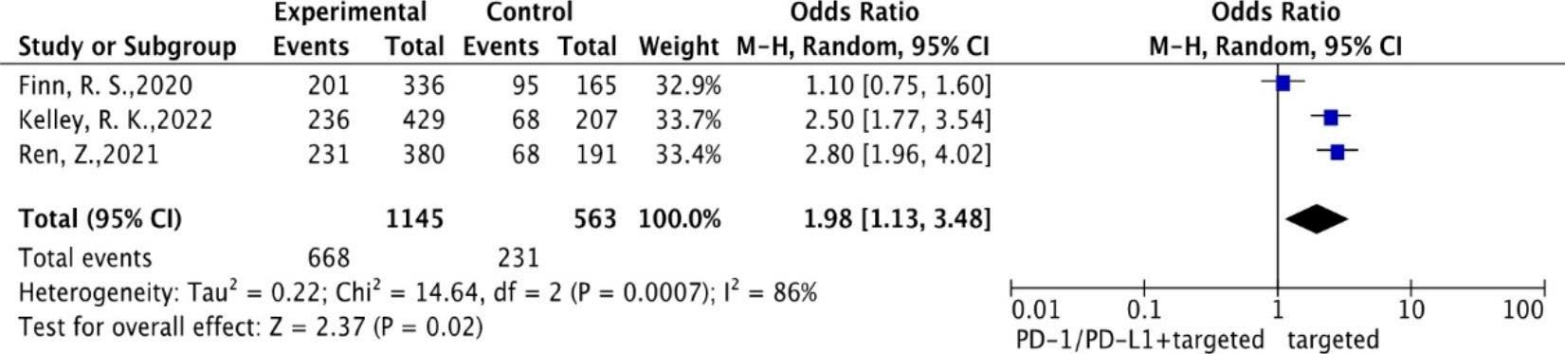
Forest plot of odds ratios of treatment-related adverse events using a random-effects model of hepatocellular carcinoma

All trials reported the incidences of grade 3–5 TRAEs. The pooled results showed that the combined therapy was associated with a significantly higher incidence of grade 3–5 TRAEs compared with targeted therapy alone (OR = 1.98; 95% CI: 1.13–3.48) (Fig. 5).

## Discussion

Cancer treatment in unresectable HCC and other malignant solid tumors has been rapidly changing, and the combinational therapy is increasingly favored. We performed a systematic review and meta-analysis to provide targeted therapy in combination with PD-1/PD-L1 inhibitors compared with targeted monotherapy. Our analyses explored clinically relevant efficacy outcomes, including OS, PFS, and grade 3–5 TRAEs. According to the results of the present study, targeted therapy in combination with PD-1/PD-L1 checkpoint inhibitors significantly improved the OS and PFS for unresectable HCC compared with targeted monotherapy showed that for unresectable HCC.

Three phase III RCTs comparing targeted therapy in combination with PD-1/PD-L1 inhibitors with targeted monotherapy have been published so far. Finn et al. reported the combination therapy of atezolizumab (anti-PD-L1) and bevacizumab, which is a vascular endothelial growth factor A (VEGF-A) inhibitor, as compared to sorafenib targeting anti-angiogenesis multikinase receptor, with statistically significant and clinically meaningful improvement in both OS and PFS in the treatment of unresectable HCC [14]. Ren et al. and Kelley et al., respectively, reported that sintilimab (anti-PD-1) plus bevacizumab biosimilar and atezolizumab plus cabozantinib, which is a tyrosine kinases c-Met and VEGFR2 inhibitor, compared with sorafenib, achieved clinically meaningful improvements in OS and PFS for advanced/unresectable HCC [15, 17].

In patients with unresectable/advanced HCC, to the best of our knowledge, this is the first meta-analysis on RCTs to investigate the efficacy of targeted therapy in combination with immunotherapy versus targeted monotherapy. Although several trials are still ongoing, only three RCTs have been published. Compared with sorafenib, significantly better OS and PFS were observed with sintilimab plus bevacizumab (HR = 0.57, 95% CI: 0.43–0.75; HR = 0.56, 95% CI: 0.45–0.69), and atezolizumab plus bevacizumab (HR = 0.58, 95% CI: 0.58–0.80; HR = 0.59, 95% CI: 0.46–0.75), respectively. In terms of grade 3–5 AEs, the uses of lenvatinib (HR = 1.51; 95% CI: 1.14–2.00) and linifanib (HR = 1.94, 95% CI: 1.41–2.66) were higher than sorafenib. More data from updated clinical trials are still needed to confirm the benefit of combination therapy for HCC patients.

In the analyses of TRAEs, the results showed that compared with targeted monotherapy, the combination therapy had a significantly higher incidence of grade 3–5 TRAEs. The most common grade 3 or 4 TRAE with atezolizumab + bevacizumab and sintilimab + bevacizumab biosimilar group was hypertension (both 15%), which is consistent with the established safety profile of bevacizumab [18]. Besides, gastrointestinal disorders were the most common reasons for treatment discontinuation (5%) in both groups, as expected in patients with liver cancer and underlying cirrhosis. The most common grade 3 or 4 TRAE was alanine aminotransferase increase (9% in the cabozantinib plus atezolizumab combination treatment group). In one of the included studies, Kelley et al. reported immune-mediated adverse events of any grade requiring immunosuppressive treatment occurred in 31 (7%) of 429 patients in the combination treatment group [17]. The most common ones were hepatitis (4%) and pneumonitis (2%). For these 3 trials, the most common TRAEs from targeted agents were hypertension and elevated alanine aminotransferase. Grade 3 or 4 TRAEs, immune mediated or non-immune mediated, leading to study treatment discontinuation were infrequent in these 3 trials, indicating that these TRAEs were manageable with immunosuppressive drugs or other treatments. Potential candidates for the combination therapy of targeted drug and immunotherapy should be provided with this information.

Unresectable HCC management is still challenged in patients with cirrhosis and varied degree of impaired liver function. Immunotherapy, such as pembrolizumab and nivolumab, has been a viable and safe option in patients with advanced HCC [19]. Newer systemic drugs like the combination of immune checkpoint inhibitors with biologic therapy, such as ramucirumab plus durvalumab treatment, likely to be promising as a new treatment standard for patients with unresectable HCC [20].

Atezolizumab is also used for other cancers like non-small cell lung cancer and advanced renal cell carcinoma. The COSMIC-021 study reported the combination therapy with atezolizumab and cabozantinib for advanced renal cell carcinoma [21], which regimen was similar to COSMIC-312 trial [17], appeared to be tolerable with a manageable toxicity profile. Grade 3 or 4 TRAE occurred in 59% of patients, slightly higher than 36% of patients of COSMIC-312 trial. Grade 3 or 4 Immune-mediated events were 30%. TRAEs leading to discontinuation of drug was 19–24% for subgroups. All AEs were managed with dose modifications and supportive care.

As for the second-line treatment, regorafenib showed promising results after sorafenib failure in HCC patients [22]. One meta-analysis evaluating the efficacy and safety of regorafenib in unresectable HCC showed that pooled objective response rate was as high as 10.1% and pooled median OS of 11.1 months, as well as TRAE greater than Grade 3 was 50-58.3%. Regorafenib represents a valuable and comparatively safe therapeutic option in patients who progress on sorafenib [23]. HCC scenario is continuously and rapidly changing for decades due to different etiology and treatment advance, including the progressions of patients age, increased non-viral cases and an earlier stage migration [24]. This molecular information should also be integrated in the future to guide us to deal with the cancer more precisely [25].

The present study included several limitations. First, this meta-analysis mainly compared combination therapy with targeted monotherapy. These included RCTs, however, used various targeted agents and immunotherapy drugs, which may have biased the data analysis from the dissimilar therapeutic effects and AEs between drugs. The efficacy and TRAEs of individual drug in the combination can be further investigated using indirect comparison in the future. Second, the comparison of combination therapy with targeted monotherapy in patients with advanced/unrespectable HCC included only three RCTs with the limited information. Some other ongoing studies, such as LEAP-002 (lenvatinib plus pembrolizumab versus lenvatinib) [26] may be included in the near future. Third, the cost-effective analysis was insufficient in these trials. Cost-effective issue for HCC might be important because of the higher cost for the combination therapy than targeted monotherapy. More studies, especially those with the cost-effective analysis, are warranted in the future.

## Conclusion

Our meta-analysis concluded that compared with targeted monotherapy, targeted therapy in combination with PD-1/PD-L1 inhibitors provided the survival benefits in patients with unresectable HCC. The patients receiving combination therapy had significantly higher incidences of grade 3–5 adverse effects.

## Data Availability

The datasets used and/or analysed during the current study are available from the corresponding author on reasonable request.
